# Novel approach to determination of sorption in pervaporation process: a case study of isopropanol dehydration by polyamidoimideurea membranes

**DOI:** 10.1038/s41598-017-08420-0

**Published:** 2017-08-21

**Authors:** A. Pulyalina, G. Polotskaya, M. Goikhman, I. Podeshvo, B. Chernitsa, V. Kocherbitov, A. Toikka

**Affiliations:** 1 Saint-Petersburg State University, Department of Chemical Thermodynamics & Kinetics, Saint-Petersburg, 198504 Russia; 2 Institute of Macromolecular Compounds, Russian Academy of Sciences, Saint-Petersburg, 199004 Russia; 3 Biomedical Science, Faculty of Health and Society, Malmö University, Malmö, SE-205 06 Sweden

## Abstract

Development of novel membranes with optimal performance, selectivity, and stability is a key research area in membrane technology. In the present work aromatic polyamidoimideurea (PAIU) is synthesized and tested as promising membrane material for separation of water and alcohol mixtures. The PAIU membrane structure, density, and transport properties are studied. Mass transfer of water and isopropanol through the membrane is estimated by sorption and pervaporation tests to determine equilibrium sorption degree, diffusion coefficients, flux through the membrane, and separation factor. Two techniques of sorption study from liquid and from vapor phases are used as novel approach to experimental study of mass transfer. The vapor sorption calorimetry permits to analyze the behavior of the polymer material in sorption process. In pervaporation of water–isopropanol mixture, almost pure water mainly permeates through PAIU membrane. To improve the performance, a double layer membrane containing a thin PAIU layer on the surface of porous poly(phenylene oxide) support is developed. The double layer membrane is extremely effective in dehydration of isopropanol.

## Introduction

Membrane technologies have found its application in industrial and ecological processes due to their operational simplicity, low power consumption, modular and compact equipment as compared to physical and chemical analogues^1, 2^. Pervaporation is a membrane process that allows separating the components of the liquid mixture by transfer through the membrane by permeation and vaporization^3^. The membrane acts as a selective barrier between the two phases - the liquid phase of feed and the vapor phase of permeate. The mass transfer through the membrane occurs by the mechanism of “solution-diffusion”^4^ with the following stages: adsorption of a penetrating component on the membrane surface, dissolution of a component in the membrane material, diffusion of the component through the membrane and desorption from the back side of the membrane. Transport of small molecules through the polymer membrane can be described by the equation: *P* = *D* · *S*, where *P* is the permeability coefficient, *D* is the diffusion coefficient, and *S* is the solubility coefficient. In pervaporation the limiting stage that determines the intensity of the transfer is sorption and dissolution of the components in the membrane material; solubility is a thermodynamic constituent of the mass transfer process^5^.

Pervaporation process is widely used in separation of various liquid mixtures such as solutions with similar boiling points, thermally sensitive compounds, including organic‒organic and water‒organic mixtures with azeotropic point^6^. Separation of water–isopropanol mixture is one of the known applications of pervaporation^7–9^. The isopropanol is widely used as a cleaning agent in modern chemical, semiconductor, and electronic industries. Dehydration of wasted isopropanol is essential from environmental and economical points of view. The existence of water–isopropanol (12.2: 87.8 wt%) azeotropic mixture causes difficulties in isopropanol recovery by conventional distillation. Pervaporation does not require any chemicals addition to effective separation of water–alcohol mixture; it is considered as a prospective approach to outperform conventional separation technologies.

Modern industrial tasks stimulate the development of the advanced membranes with improved transport properties. Polymers are the most versatile and feasible materials among diverse choices of membrane materials^10^. Efficiency of polymer membrane materials depends on numerous factors: the chemical nature of macromolecules, physicochemical properties and structure of membrane, properties of separating mixture, etc.

Extensive research has been done in finding the optimal polymer membrane that has maximal performance such as selectivity, flux, and stability. Polymers of heteroaromatic structure are known to exhibit specific physical and chemical properties such as increased structural order and glass transition temperature, fixed free volume, and thermodynamic parameters^11–13^. Membranes based on polyimides, polyamidoimides, and polyetherimides have been effective in separating of water–alcohol mixtures by pervaporation^14–20^. Polybenzimidazoles have been applied for the dehydration of organic solvents^21^.

Many characteristics including transport properties of these polymers depend on the prehistory of the membrane preparation. The macromolecules of heteroaromatic structure exhibit tendency to the formation of the donor-acceptor bonds with amide solvents (dimethylformamide, *N*-methylpyrrolidone, etc) used in their synthesis and in the following membrane preparation. The macromolecule–solvent complexes are so stable that they do not destruct during transition to the solid state. This fact prevents the total removal of the solvent from the samples by long drying at 80 °C in vacuum. The presence of residual solvent greatly influences the transport parameters of the membranes^22–26^.

Polymers of heteroaromatic structures exhibit relatively low permeability in diffusion processes. High permeability can be achieved by formation of the composite membrane containing selective polymer in the form of thin top layer arranged on the surface of a porous support that ensures mechanical strength of the membrane^27, 28^. Composite membrane with ultra-thin top layer of polyamide on polytetrafluorethylene porous support showed high productivity in the separation of water–isopropanol mixture^29^. Composite membranes containing ultra-thin polyamide layer on a modified polyacrylonitrile support were effective in pervaporation of aqueous solutions of ethanol and isopropanol^30^. The separation of water–isopropanol (70:30 wt%) mixture at 70 °С occurred with the significant flux and more than 99 wt% water concentration in permeate.

**Figure 1:**
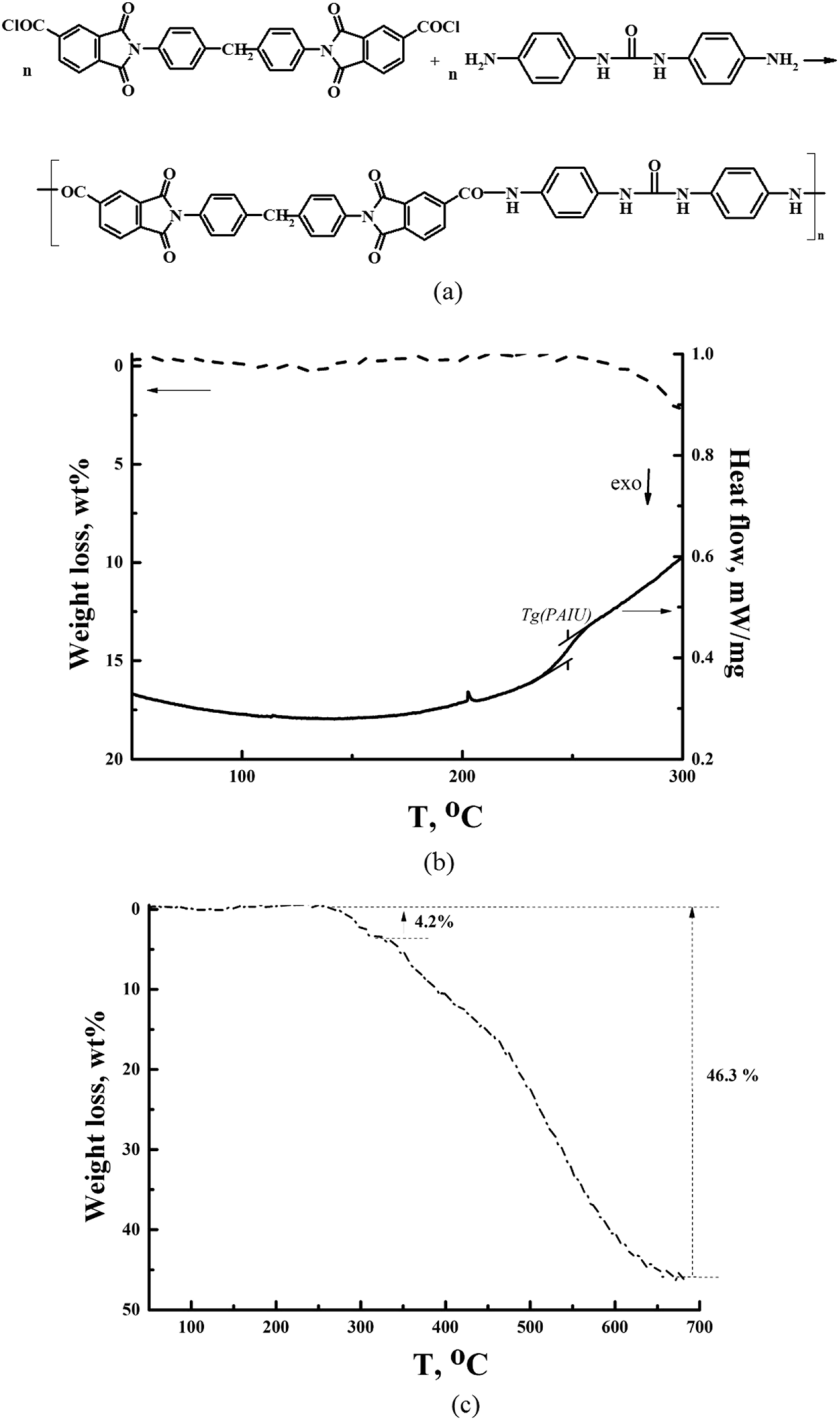
Scheme of PAIU synthesis (**a**), TG (**b**,**c**) and DSC (**b**) curves of PAIU.

The object of the present work is a polymer of heteroaromatic structure containing imide and amide groups in combination with urea groups in the backbone, so called polyamidoimideurea (PAIU). It can be expected an influence on sorption activity of functional groups in the structure of the monomer unit. The aims of the work are *i)* to synthesize PAIU (Fig. 1a) and to prepare dense films and composite membranes based on PAIU and *ii*) to study transport properties of the membranes in the pervaporation of a water–isopropanol. The mass transfer in pervaporation depends mainly on the thermodynamic factors responsible for the sorption of penetrant by membrane at equilibrium. Therefore, much attention was paid to the sorption tests of dense films in the medium of water and isopropanol as components of separating mixture.

## Results

Physical properties of PAIU film were determined. The value of water contact angle equal to 78.6° ± 0.1° shows that the surface of the PAIU film is wetted. The PAIU film exhibits the density equal to 1.359 ± 0.006 g/cm^3^ that is typical for polymers of heteroaromatic structure.

Thermal stability and glass transition temperature of PAIU film were characterized by TGA and DSC (Fig. 1b and c). The polymer under study is thermally stable and its degradation begins over 350 °C. The first range of weight loss from 250 °С to 320 °С reflects the removal of residual solvent NMP and the destruction of the amide groups of the polymer chains. The total weight loss above 46.3 wt% is observed at 680 °C. Fig. 1b shows DSC data; the second heating cycle of DSC was used to determine glass transition temperature. T_g_ of PAIU is equal to 243 ± 2 °C.

Transport properties of PAIU films were estimated for the mass transfer of water and isopropanol mixture. Sorption degrees of water and isopropanol and diffusion coefficients of the penetrants were determined in sorption tests, whereas the PAIU membrane performance and separation factor were determined in pervaporation.

### Sorption Study

**Figure 2:**
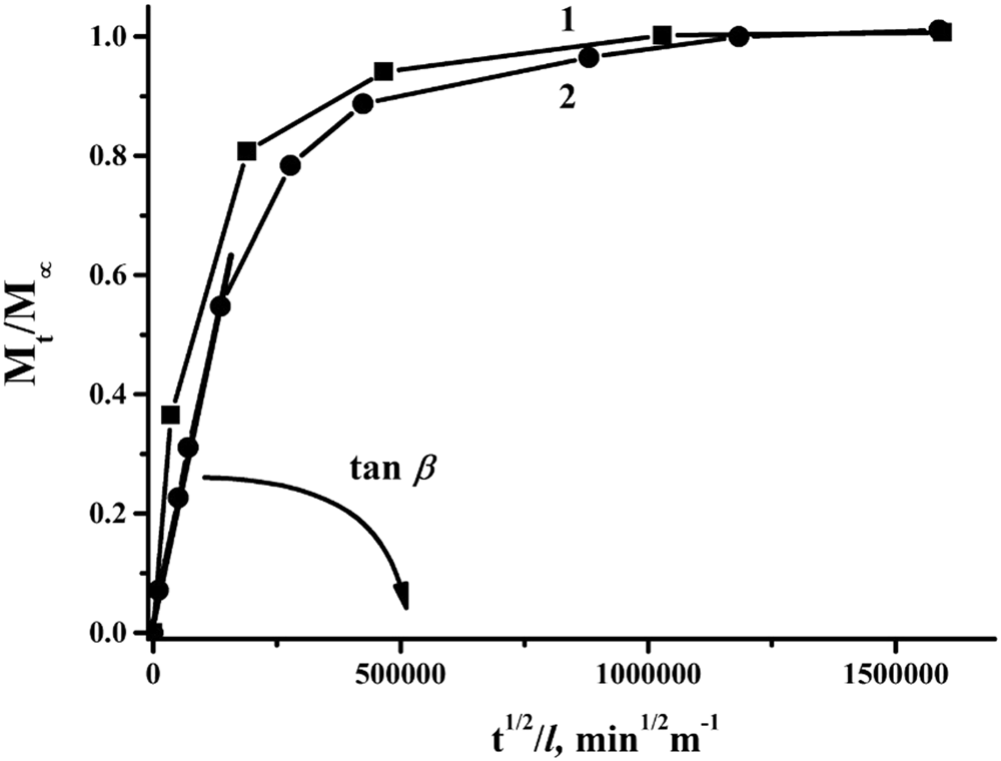
Kinetic curves of (1) sorption and (2) desorption of water in PAIU film.

The series of sorption and desorption tests were made to estimate sorption and diffusion parameters of water and isopropanol in PAIU film. Sorption tests were performed by immersing the samples into individual pure liquids. Desorption tests were carried out at isothermal and isobaric conditions. It was found that the PAIU film exhibits better sorption of water as compared to isopropanol. Presence of amide and urea functional groups in the PAIU backbone facilitates sorption of water molecules by hydrogen-bonding. Figure 2 shows kinetic curves of the water sorption and desorption. The value of the isopropanol sorption in the membrane was small, which did not allow plotting the sorption/desorption curves and determining the diffusion coefficient of isopropanol.

The diffusion coefficient of water in the film was estimated based on the Fick’s second law. The kinetic curves of the desorption (Fig. 2) was employed to calculate the diffusion coefficient, where *M*_*t*_ is the amount of desorbed substance per time *t*. The linear part on the desorption curve corresponds to the system obeying the second Fick’s law and enables to calculate the effective diffusion coefficients of liquids. The last parameter characterizes the penetration rate of the liquid molecules and influences on the separation efficiency of the membrane in the pervaporation.

The kinetic of sorption and desorption processes of PAIU membrane and the linear part on the curve for diffusion coefficient calculation are demonstrated on Fig. 2.

Calculated sorption and diffusion parameters are presented in Table 1. The sorption degree of water significantly exceeds the same parameter of isopropanol. The diffusion ability of water is also much higher as against isopropanol due to difference in molecular size of water and alcohol. This fact determines the perspective use of the PAIU membrane in alcohol dehydration by pervaporation.

**Table 1:** The sorption degrees and the diffusion coefficients of the penetrants in PAIU film.

Liquid	Sorption degree, %	Diffusion coefficient, m^2^/min
Water	6.80 ± 0.02	(1.01 ± 0.03) · 10^−11^
Isopropanol	0.69 ± 0.04	(5.90 ± 0.09) · 10^−13^

**Figure 3:**
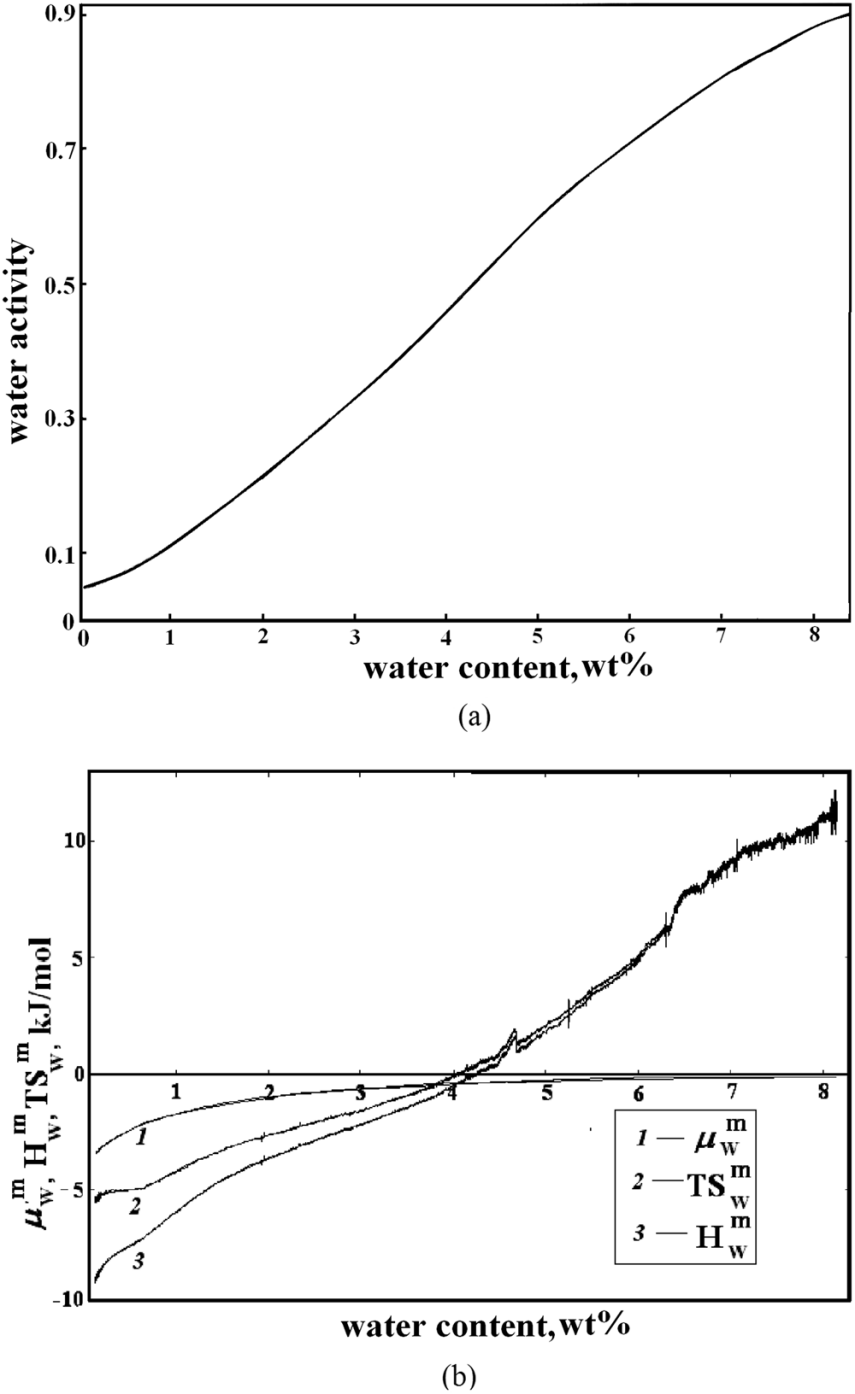
Sorption isotherm of water (**a**) and the partial molar Gibbs energy ${{\rm{\mu }}}_{{\rm{w}}}^{{\rm{m}}}$, enthalpy ${H}_{{\rm{w}}}^{{\rm{m}}}$, and entropy ${S}_{{\rm{w}}}^{{\rm{m}}}$ of water mixing (**b**) for PAIU film.

The interaction between the polymer and water was studied in details by vapor sorption calorimetry at 25 °C. The water sorption isotherm of PAIU is presented on Fig. 3a. The sorption isotherm has a sigmoidal shape with a point of inflection. At lower water activity (up to 4 wt% of water) the isotherm is concave towards the *y*-axis and at higher activity the isotherm has the opposite trend. The partial molar Gibbs energy ${{\rm{\mu }}}_{{\rm{w}}}^{{\rm{m}}}$, enthalpy ${H}_{{\rm{w}}}^{{\rm{m}}}$ and entropy ${S}_{{\rm{w}}}^{{\rm{m}}}$ of mixing of water in PAIU were measured simultaneously with the sorption isotherm and are presented in Fig. 3b.

The results of sorption calorimetric experiments (sorption isotherm of water and calculated partial molar Gibbs energy, enthalpy and entropy of water mixing for PAIU film) are presented on Fig. 3.

At low water contents, the partial enthalpy of mixing of water with the sample is about −12 ± 0.1 kJ per mole of water (exothermic effect). This shows that at low water contents the film is in the glassy state. The exothermic heat effect arises from the loss of mobility of water during transport from liquid water to the solid glassy matrix of the polymer, where water molecules lose some degrees of freedom. This behavior was observed before in various systems that are able to form a glassy amorphous state^31–33^.

At higher water contents, the hydration enthalpy became endothermic. It is known that the values of enthalpy of mixing indicate the character of the polymer–penetrant interaction related to the structural features of polymer. The polymer chains become more flexible due to the fact that water acts as a plasticizer and at gradual transition to the elastic state occurs. For the PAIU film the isothermal glass transition is observed at water content of about 4 wt% at 25 °C.

### Pervaporation using PAIU membrane

**Figure 4:**
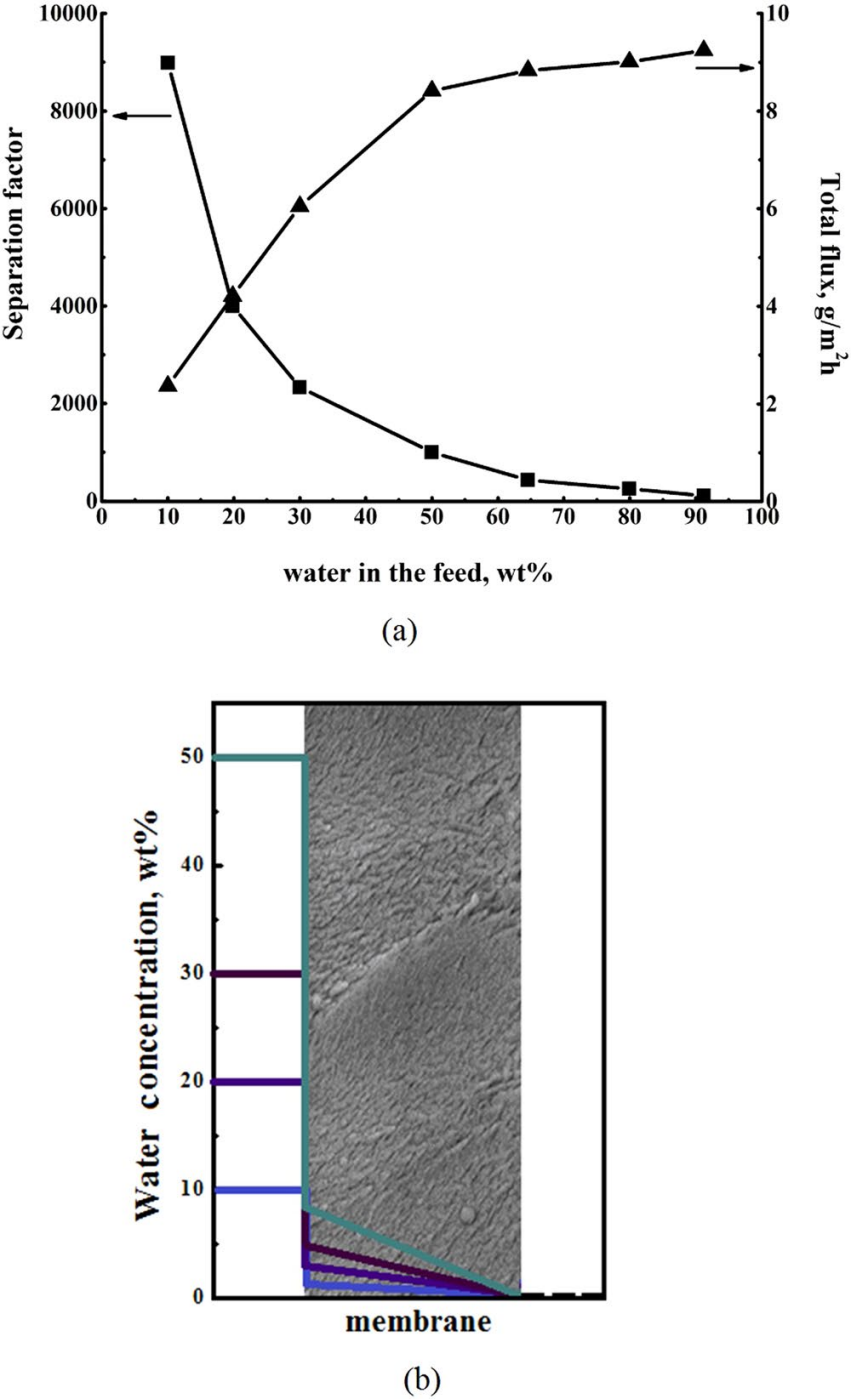
The dependence of separation factor and total flux on the feed composition in pervaporation of water–isopropanol (**a**) and the calculated concentration profile of water through the PAIU membrane (**b**).

The performance of PAIU membrane in the separation of a water–isopropanol mixture was studied in a wide range of feed compositions. Figure 4a shows the dependence of the separation factor and the total flux on the water concentration in feed. The PAIU membrane is mainly permeated by water and it exhibits high values of separation factor *α*_*water*/*IPA*_ especially in the range of small water concentration in the feed. The fact that sorption and diffusion properties of water are greater than that of isopropanol provides high selectivity of PAIU in separation of water–isopropanol mixture.

Data of pervaporation performance (separation factor and total flux) of PAIU membrane and concentration profile of water at the membrane surface as preferably permeate component of feed mixture are shown on Fig. 4.

The total flux through PAIU membrane increases with the rise of water concentration in feed. The value of total flux is corresponding to a moderate value of fluxes for polymers of heteroaromatic structure.

The sorption isotherm of water (Fig. 3a) was used for determination of the bulk concentration profile at the membrane surface (${C}_{{i}_{0}}^{f}$). The water activities in feed mixtures under study (10, 20, 30 and 50 wt% of water) were calculated using vapor-liquid equilibrium data of water-isopropanol system. Then, from the water activity, using the sorption isotherm, the concentration of water in the membrane was obtained (Table 2). The concentration profile through the PAIU membrane is shown on Fig. 4b.

**Table 2:** The bulk water concentration at the surface diffusion coefficients of water in pervaporation for PAIU membrane.

Water concentration in the feed, wt%	Water activity in the feed,	Bulk water concentration at the surface, wt%	Diffusion coefficient · 10^−11^, m^2^/min
10	0.2	1.9 ± 0.03	0.60 ± 0.01
20	0.4	3.7 ± 0.07	0.64 ± 0.03
30	0.6	5 ± 0.06	0.68 ± 0.02
50	0.88	8 ± 0.02	0.70 ± 0.01

To understand the mechanism of molecular transport in pervaporation over time, Fick’s first law was employed. The diffusion ability of the preferably permeating component (water) was estimated by the calculation of the diffusion coefficients *D*_*i*_ based on Equation (1):

1 $${D}_{i}=\frac{J\cdot l}{{C}_{{i}_{0}}^{f}-{C}_{il}^{p}}\approx \frac{J\cdot l}{{C}_{{i}_{0}}^{f}}$$

where *J* is the flux per unit area (g/m^2^ min), *D*_*i*_ is the diffusion coefficient (m^2^/min), ${C}_{{i}_{0}}^{f}$ is the bulk concentration at the membrane surface of component *i* on the feed side (g/m^3^), *l* is membrane thickness, m. Value of ${C}_{il}^{p}$ is neglected due to the concentration of the component *i* on the surfaces on the permeate side is zero. The results are presented in Table 2.

To improve the performance of isopropanol dehydration in pervaporation, double layer membranes composed of PAIU thin selective layer on a surface of porous PPO film was developed. A porous PPO film was chosen as a support because it has been successfully used for the production of composite membranes with a selective layer that consists of polymers with heteroaromatic structure^34–36^.

**Figure 5:**
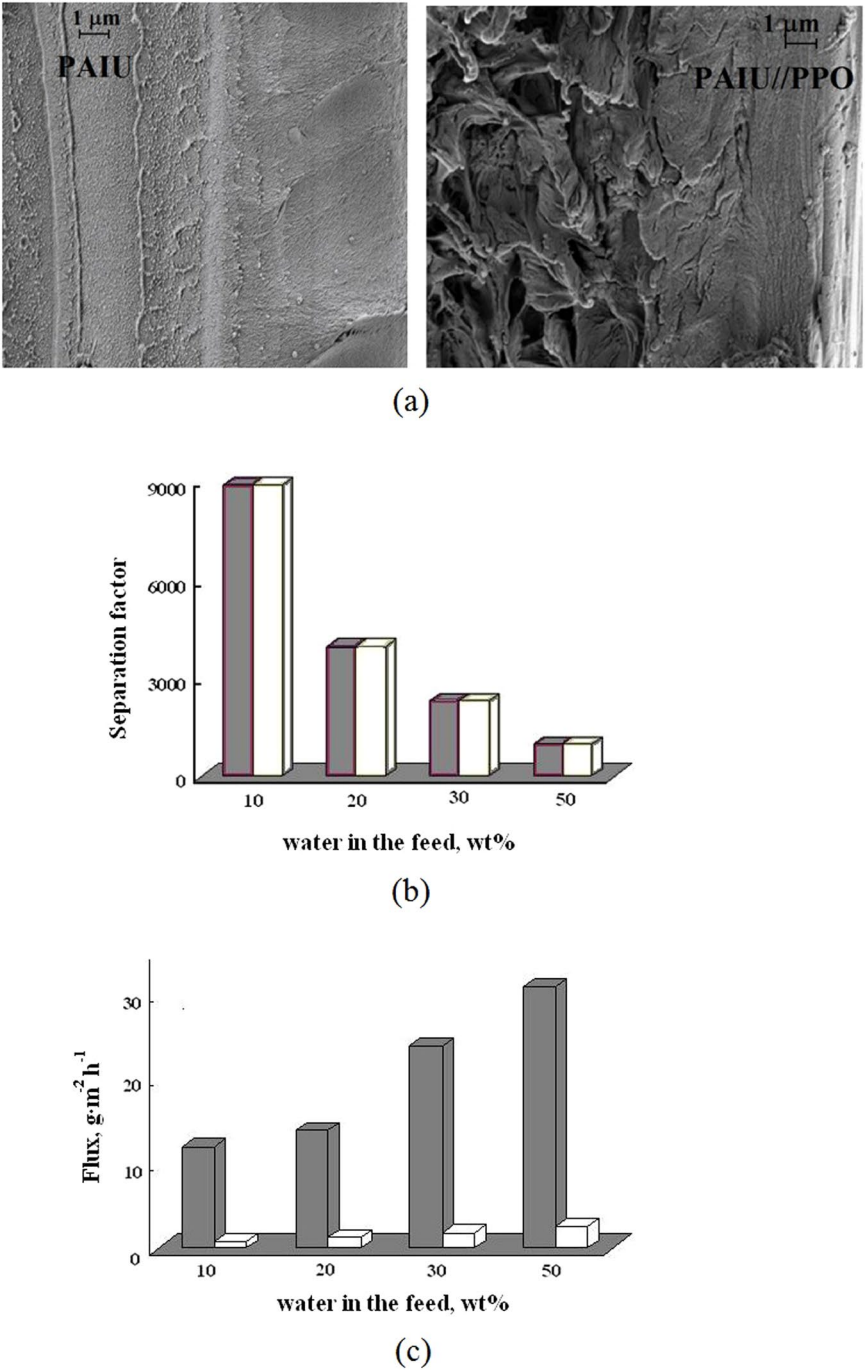
The SEM micrographs on cross-section of PAIU and PAIU/PPO films (**а**), the dependence of separation factor (**b**) and total flux (**c**) on the water content in the feed in pervaporation of water–isopropanol mixture using () PAIU and () PAIU/PPO membranes.

The morphology of the PAIU/PPO membrane was investigated by SEM. Figure 5a shows the micrographs of cross-section of two type membranes based on PAIU.

Characterization of double layer PAIU/PPO membrane: structure characterization by SEM and the effectiveness of PAIU/PPO in pervaporation separation are presented on Fig. 5.

PAIU membrane has a dense homogeneous cross-section with small elements of supramolecular structure. The micrograph of the PAIU/PPO demonstrates the uniform structure of the dense PAIU top layer (on the right) and a part of the porous PPO support exhibited a spongy structure. Figure 5a demonstrates the defect-free PAIU top layer with thickness approximately equal to 4–5 μm.

The performance of PAIU/PPO membrane was studied in pervaporation of a water–isopropanol mixture in a wide range of the feed composition including the azeotropic point. Figure 5b and c were plotted to compare transport properties of two type membranes in pervaporation of four feed composition. Figure 5b demonstrates high values of separation factor for PAIU/PPO membrane that are similar to that of PAIU membrane.

Figure 5c shows the significant rise of the total flux in the case of PAIU/PPO membrane. For each feed composition the total flux through PAIU/PPO membrane is more than an order of magnitude greater than the flux through PAIU membrane of 20 μm thickness.

### Comparison of transport properties of the present membranes with literature data

The transport properties of PAIU membrane developed in this work were compared with literature data for the case of pervaporation separation of the isopropanol‒ water mixture in composition closed to azeotropic point. Table 3 lists data on operating temperature and feed concentration as well as membrane characteristics in pervaporation: separation factor and total flux that have been obtained for different polymer membranes in a number of published works^8, 9, 37–41^. The value of the separation factor of the PAIU membrane exceeds that of many previously published reports.

**Table 3:** Comparison of transport properties of membranes in pervaporation of isopropanol‒water mixture.

Membrane	Temperature, °С	Water concentration in feed, wt%	Separation factor	PSI	Ref.
PAIU/PPO	50	10	9000	135000	Present work
P84®	60	15	16	41248	8
P84 (cross-linked with ethylene diamine)	60	15	604	322536	8
Ultem®	60	15	585	4095	9
Polyesterimide	50	12	270	297	51
Polyesterimide/co-polyaniline(5%)	50	12	400	2600	51
Matrimid®	25	14	2 000	2400000	52
Poly(benzoxazole-co-imide)	60	15	2826	5934600	53
Polybenzoimidazole	25	10	4410	123480	54
Polybenzoimidazole (chitosan-modified)	25	20	218	32700	54
Polyimide (BPADA–8ODA–2DABA)	60	10	2243	100935	55

## Discussion

Physicochemical and transport properties of poly(4,4′-diaminodiphenylcarbamide)-4,4′-dicarboxydiphenylmethane in the form of PAIU membranes were studied. Combination of imide, amide, and urea groups in the backbone influences PAIU physicochemical and transport properties. The measured value of water contact angle indicates the hydrophilic nature of the surface of PAIU membrane. Mass transfer of water and isopropanol through PAIU membrane was studied by sorption and pervaporation tests to determine main transport parameters: sorption degree, diffusion coefficients, flux through the membrane and separation factor.

In pervaporation of water–isopropanol mixture, the PAIU membrane is mainly permeated by water and possesses high separation factor towards water in a wide range of feed composition.

Novel approach was proposed for mass transfer study which consists in using two techniques of sorption: from liquid and from vapor phases. The additional technique of vapor sorption calorimetry permits to analyze the behavior of the polymer material in sorption process. Sorption isotherms were obtained and used to model the water concentration profile through the membrane. The developed algorithm based on Fick’s first law can be regarded as alternative method for calculating diffusion coefficients in the membrane.

The PAIU membrane has higher sorption affinity and diffusion ability of water in comparison with isopropanol. Thus the transport properties of membrane mainly determine the water permeability through the membrane. At low water concentration in the feed the water concentration at the membrane surface is even lower and increases with the increase of water concentration in separating mixture (Fig. 4b). The same trend is observed for the calculating diffusion coefficient of water (Table 2). The increase of the permeation coefficient of water promotes the rise of flux through the membrane at the separation of mixtures with high water concentration (Fig. 4a). It should be mentioned that the values of diffusion coefficients calculating based on Fick’s first law (the Equation 1 and Table 1) are in agreement with the value of the diffusion coefficient estimated based on Fick’s second law using sorption/desorption experiments for pure water (1 · 10^−11^ m^2^/min).

The double layer composite membrane consisting of PAIU selective top layer on porous PPO film support also exhibits high selectivity in pervaporation of water–isopropanol mixture. The flux through the PAIU/PPO membrane is a few times greater as compared to the PAIU membrane in the process of the dehydration of an aqueous solution of isopropanol for all feed compositions.

## Methods

### Materials

*N*-methylpyrrolidone (NMP), propylene oxide, methanol, and isopropanol of chemically pure (CP) grade were purchased from Vekton (Russia) and were used without further purification. 4,4′-diaminodiphenylurea was were synthesized as described in^42^. *N*,*N*′-diphenylmethane-*bis-* (trimellitimido)acid was synthesized as described in ref. 43.

### Polymer synthesis

Poly (4,4^/^-diaminodiphenylcarbamide)-4,4^/^-N,N′-diphenylmethane-*bis-*(trimellitimido) carboxylate (polyamidoimideurea, PAIU) was synthesized by low temperature polycondensation (Fig. 1). 4,4^/^-diaminodiphenylurea (0.001 mol) and NMP (8 mL) were placed in a three-neck round bottom flask with a stirrer and a thermometer. After dissolution the flask was cooled to −15 °С and then dichloroanhydride of *N*,*N*′*-*diphenylmethane-*bis*(trimellitimido) acid (0.001 mol) was added. The stirring was continued for 1 h, after that the cooling bath was removed. Then 1–2 drops of propylene oxide were added and the stirring was continued at room temperature for 5 h. The obtained transparent polymer solution was filtered and used for membrane preparation.

### Membrane Preparation

PAIU films (20–35 μm thickness) were prepared by casting of PAIU solution in NMP on glass plate followed by evaporation of solvent at 80 °С in air and drying to a constant weight at 80 °С in vacuum. Then dense nonporous PAIU films were subjected to a special treatment by immersing in methanol for 24 h followed by drying at 50 °С in vacuum to constant weight.

PAIU/PPO double layer membranes were prepared by casting PAIU solution in NMP on the surface of porous poly(2,6-dimethyl-1,4-phenylene oxide) (PPO) support that was obtained as described in refs 44, 45. PAIU/PPO films were dried at 80 °С in air and finally in vacuum.

### Characterization procedures

Thermogravimetric analysis (TGA) was conducted using samples of ~10–14 mg. They were contained in a platinum crucible with a heating speed of 10 deg/min in a nitrogen atmosphere. The TG 209 F3 Iris thermo-microbalance (Netzsch) was used for the analysis.

The glass transition temperature (*T*_*g*_) was determined using differential scanning calorimeter DSC 204 F1 (Netzsch, Germany). The analysis was conducted under inert atmosphere with samples of approximately 4–5 mg at a scan rate of 10 °C/min from −20 to 300 °C.

Membrane morphology was studied by scanning electron microscope SEM Zeiss SUPRA 55VP. Before the test the gold layer was coated on the sample surface by cathode sputtering using the Quorum 150 (Great Britain) installation.

### Contact angles measurement

The wettability of the films by water was estimated by measuring the contact angle of water on the film surfaces by sessile drop method on the Drop Shape Analyzer DSA 10 (KRÜSS, Germany) under atmospheric pressure and temperature 20 °C. Water was used as test liquid (the surface tension is 72.4 mN/m).

### Density Determination

The membrane density was estimated by the flotation method with a laboratory made measurement unit^46^. The mixture of toluene and carbon tetrachloride was used to equilibrate the specimens at 20 °C.

### Sorption Experiments

Sorption of water or isopropanol from liquid phase was tested in the following way. Samples of PAIU film were completely immersed in a bottle containing the solvent under study. The bottle was placed inside a thermostat at a constant temperature of 20 °C. After defined time intervals, the samples were blotted using filter papers to electronic balance (Shinko HTR-220CE) with an accuracy ±0.0001 g. Then the samples were placed back in the same solvent. The experiment was continued until the maximum value of sorption (equilibrium sorption) was reached.

After completion of sorption experiments, the solvent desorption was carried out by exposing the samples in the air atmosphere of the desiccator containing zeolites. The weight changes as a function of time were measured. Finally, a sorption curve that shows an uptake of a solvent versus square root of time was plotted and analyzed to determine the diffusion parameters of the PAIU film.

The equilibrium sorption degree (*S*) was calculated by equation

2 $$S=\frac{{m}_{s}-{m}_{d}}{{m}_{d}}\cdot 100 \% $$

where *m*_*s*_ is the weight of a film sample in sorption equilibrium state and *m*_*d*_ is the weight of a dry sample.

The sorption and desorption of a solvent in glassy polymer films is affected by the solvent–polymer interaction and its kinetics can be divided into two parts: Fickian sorption and further sorption contributed by the relaxation of the polymer matrix. The sorption kinetics conforming to the criteria required for Fickian type of sorption can be described by the following equation based on the Fick’s second law

3 $$\frac{{M}_{t}}{{M}_{\infty }}=1-\frac{8}{{\pi }^{2}}\sum _{n=0}^{n=\infty }\frac{1}{{(2n+1)}^{2}}\,exp[\frac{-D{(2n+1)}^{2}{\pi }^{2}t}{{l}^{2}}]$$

or by another form of this equation used for the initial period of time

4 $$\frac{{M}_{t}}{{M}_{\infty }}=\frac{4}{{\pi }^{1/2}}{(\frac{Dt}{{l}^{2}})}^{1/2}$$

where *D* is diffusion coefficient, *l* is the thickness of a dry polymer film, *n* is an integer number, *M*_*t*_ is the weight gain at time *t*, and *M*_*∞*_ is the equilibrium uptake.

Accordingly the kinetic curves of desorption *M*_*t*_ */M*_*∞*_ = *f (t* ^*1/2*^ */l)* were plotted, where *M*_*t*_ is the amount of sorbed/desorbed substance per time *t*, *M*_*∞*_ is the equilibrium amount of substance that was determined as a difference between the weight of the swollen film and the weight of the film dried to constant weight, and *l* is the film thickness^47–49^.

The effective diffusion coefficient *D* was calculated by the equation

5 $$D=\frac{\pi }{16}{(tan\beta )}^{2}$$

where $tan\,\beta =\frac{{M}_{t}}{{M}_{\infty }}\cdot \frac{l}{{t}^{1/2}}$ is tangent of the initial linear slope of the desorption kinetic curves when *М*_*t*_/*М*_*∝*_ < 0.4.

The water vapor sorption was studied by sorption calorimetry method^50, 51^. Sorption calorimetric experiments were conducted at 25 °C in a 28 mm two-chamber sorption calorimetric cell inserted in a double-twin microcalorimeter. The samples under study were previously dried in vacuum at room temperature. After that, they were placed in the upper chamber, and pure water was injected into the lower chamber. In a sorption experiment, water evaporates from the lower chamber, diffuses through the tube that connects the two chambers, and is sorbed by the sample in the upper chamber. It was selected a narrow tube and a relatively high sample mass. This combination provided a slow diffusion of vapor resulting in a hydration process close to equilibrium conditions.

The thermal powers released in the two chambers were monitored simultaneously. The activity of water *a*_w_ in the sorption experiments was calculated from the thermal power of vaporization of water in the lower chamber^50, 51^. The partial molar enthalpy of mixing of water ${H}_{w}^{m}$ was calculated using the following equation

6 $${H}_{w}^{m}={H}_{w}^{vap}+{H}_{w}^{vap}\frac{{P}^{sorp}}{{P}^{vap}}$$

where *P* ^vap^ and *P* ^sorp^ are the thermal powers registered in the vaporization and sorption chambers, respectively, and ${H}_{w}^{vap}$ is the molar enthalpy of evaporation of pure water.

The partial molar Gibbs energy of mixing of water ${\mu }_{w}^{m}$ was obtained as

7 $${\mu }_{w}^{m}=R\,{\rm{ln}}\,{a}_{w}$$

where *a*_w_ is the water activity, *R* is the gas constant, *T* is the absolute temperature.

The partial molar entropy of mixing of water (${S}_{w}^{m}$) was calculated using the following equation

8 $${S}_{w}^{m}=\frac{{H}_{w}^{m}}{{\rm{T}}}-R\,{\rm{ln}}\,{a}_{w}$$

### Pervaporation Experiment

Pervaporation experiments were performed using the same equipment as reported previously^52^. The active membrane area was 14.8 cm^2^. The measurements were carried out at 50 °С. The permeate pressure was kept above 0.2 mbar with a vacuum pump. The permeate was collected into a liquid nitrogen cooled trap, weighted, and analyzed. The composition of permeate was determined using both chromatograph ≪Crystal 5000.2≫ (Chromatec, Russia) with thermal conductivity detector and refractometer IFR–454B2M.

The separation factor (*α*) in pervaporation of water and isopropanol (IPA) mixture was defined with absolute error of ±1–5 as follow

9 $${\alpha }_{Water/IPA}=({Y}_{Water}/{Y}_{IPA})/({X}_{Water}/{X}_{IPA})$$

where X and Y are the weight fractions of water and isopropanol in the feed and permeate, respectively.

The total flux through membrane (*J*) was determined as the amount of substance penetrated through the membrane area per time unit. To compare membranes with different thicknesses (*l*) varying from 20 to 35 µm, the value of normalized flux (*J*_*n*_) was used. *J*_*n*_ is the flux through membrane with 20 µm thickness calculated as

10 $${J}_{n}=J\cdot l/20$$

The flux was measured with absolute error of ±0.03–0.05 g · m^−2^h^−1^ for PAIU membrane and ±0.05–0.1 g · m^−2^h^−1^ for PAIU/PPO membrane.

Membrane efficiency was estimated through the pervaporation separation index (*PSI*) that was calculated by equation:

11 $$PSI={J}_{n}({\alpha }_{Water/IPA}-1)$$

Mass transport of a binary liquid mixture through a non-porous polymeric membrane is generally described by the solution–diffusion mechanism, which occurs in three steps: sorption, diffusion, and evaporation. Thus, the selectivity and flux are governed by the solubility and diffusivity of each component of the feed mixture to be separated. In pervaporation process, because of establishing the fast equilibrium distribution between the bulk feed and the upstream surface of a membrane, diffusion becomes the limiting step that controls the migration of penetrants^53, 54^. Thus it is important to estimate the diffusion (*D*_*i*_) of the penetrating molecules in PV to understand the mechanism of molecular transport.

The transport of penetrants inside the membrane can be described with the *Fick’s first law*:

12 $${J}_{i}=-{D}_{i}{{\rm{C}}}_{i}^{m}\cdot RT\frac{\partial {\mu }_{i}}{\partial x}=-{D}_{i}{{\rm{C}}}_{i}^{m}\cdot \frac{\partial \,{\rm{ln}}\,{a}_{i}}{\partial x}=-{D}_{i}{{\rm{C}}}_{i}^{m}\cdot \frac{\partial {a}_{i}}{{a}_{i}\partial x}$$

where *J*_*i*_ is the diffusion flux, *D*_*i*_ is the diffusion coefficient, ${{\rm{C}}}_{i}^{m}$ is the concentration inside the membrane, *μ*_*i*_ is the chemical potential, *a*_*i*_ is the thermodynamic activity, *x* is the position, *R* is the universal gas constant and *T* is the absolute temperature^55^.

The driving force for diffusion over a membrane is the gradient of the thermodynamic activity of the solute over the membrane, while effects within the membrane are largely reflected by the diffusion coefficient. Equation 12 is the form of Fick’s first law where the driving force of the process (*da*_*i*_/*dx*) is separated from other effects within the membrane (${D}_{i}{{\rm{C}}}_{i}^{m}$).

In the case of one–dimensional diffusion and assumption that the concentration profile along the diffusion length is linear, the diffusion coefficient of component *i* in pervaporation process *D*_*i*_ can be calculated with the following equation

13 $${D}_{i}=\frac{J\cdot l}{{C}_{{i}_{0}}^{f}-{C}_{il}^{p}}$$

where *J* is the pervaporation flux of the component *i, l* is the membrane thickness, ${C}_{{i}_{0}}^{f}$ and ${C}_{il}^{p}$ *-* are the bulk concentrations of the component *i* at the surfaces of the membrane (0 stands for the surface on the feed side and *l* - for the surface on the permeate side).

The diffusion coefficient in equation (12) is similar to the permeability - universal parameter that used to describe the permeation through gas separation membranes^56^

14 $${P}_{i}=\frac{{J}_{i}\cdot l}{{p}_{{i}_{0}}-{p}_{il}}$$

where *J*_*i*_ is a flux of gas component *i*, and *p*_*i*0_ and *p*_*il*_ are the partial pressures of component *i* on both sides of the membrane (0 stands for the surface on the feed side and *l* stands for the surface on the permeate side).

## References

[1] Baker, R. W. Membrane Technology and Applications, 3rd Edition, John Wiley & Sons, Newark, California, USA (2012).

[2] Matteucci, S., Yampolskii, Y., Freeman, B. D., Pinnau, I. Transport of Gases and Vapors in *Glassy and Rubbery Polymers* 1–47 (John Wiley & Sons, Ltd, 2000).

[3] Wolińska-Grabczyk, A., Jankowski, A. Membranes for vapour permeation: Preparation and characterization in *Pervaporation, Vapour Permeation and Membrane Distillation: Principles and Applications* 145–175 (Elsevier Ltd, 2015).

[4] Wijmans JG, Baker RW (1995). The solution-diffusion model: a review. J. Membr. Sci..

[5] Xu Y, Chen C, Li J (2007). Experimental study on physical properties and pervaporation performances of polyimide membranes. Chem. Eng. Sci..

[6] Feng X, Huang RYM (1997). Liquid separation by membrane pervaporation: a review. Ind. & Eng.Chem. Res..

[7] Pulyalina AY, Polotskaya GA, Toikka AM (2016). Membrane materials based on polyheteroarylenes and their application for pervaporation. Russ. Chem. Rev..

[8] Qiao X, Chung TS (2006). Diamine modification of P84 polyimide membranes for pervaporation dehydration of isopropanol. AIChE J..

[9] Wang Y, Jiang L, Matsuura T, Chang T-S, Goh SH (2008). Investigation of the fundamental differences between polyamide-imide (PAI) and polyetherimide (PEI) membranes for isopropanol dehydration via pervaporation. J. Membr. Sci..

[10] Yampolskii, Y., Pinnau, I., Freeman, B. D. (Eds), Materials Science of Membranes for Gas and Vapor Separation (John Wiley & Sons, New York, USA, 2006).

[11] Ohya, H., Kudryavtsev, V. V., Semenova, S. I. Polyimide Membranes: Applications, fabrications and properties (Kodansha Ltd, 1996).

[12] Qiao X, Chung TS (2005). Fundamental Characteristics of Sorption, Swelling, and Permeation of P84 Co-polyimide Membranes for Pervaporation Dehydration of Alcohols. Ind. Eng. Chem. Res..

[13] Okamoto K (1992). Vapor Permeation and pervaporation separation of water–ethanol mixtures through polyimide membranes. J. of Membr. Sci..

[14] Jiang LY (2008). Dehydration of alcohols by pervaporation through polyimide Matrimide asymmetric hollow fibers with various modification. Chem. Eng. Chem..

[15] Qiao X, Chung T-S, Pramoda KP (2005). Fabrication and characterization of BTDA-TDI/MDI (P84) co-polyimide membranes for the pervaporation dehydration of isopropanol. J. Membr. Sci..

[16] Jianga LY, Chung T-S (2010). Homogeneous polyimide/cyclodextrin composite membranes for pervaporation dehydration of isopropanol. J. Membr. Sci..

[17] Kim J-H, Chang B-J, Lee S-B, Kim SY (2000). Incorporation effect of fluorinated side groups into polyimide membranes on their pervaporation properties. J. Membr. Sci..

[18] Lee KR, Liaw DJ, Liaw BY, Lai J-Y (1997). Selective separation of water from aqueous alcohol solution through fluorine-containing aromatic polyamide membranes by pervaporation. J. Membr. Sci..

[19] Lai JY, Li S-H, Lee K-R (1994). Permselectivities of polysiloxaneimide membrane for aqueous ethanol mixture in pervaporation. J. Membr. Sci..

[20] Jiang YW, Wang Y, Chung T-S, Qiao XY, Lai J-Y (2009). Polyimides membranes for pervaporation and biofuels separation. Prog. Polym. Sci..

[21] Wang Y, Chung TS (2010). Pervaporation dehydration of ethylene glycol through polybenzimidazole (PBI)-based membranes. 1. Membrane fabrication. J. Membr. Sci..

[22] Shau L, Chung T-S, Wensley G, Goh SH, Pramoda KP (2004). Casting solvent effects on morphologies, gas transport properties of a novel 6FDA/PMDA–TMMDA copolyimide membrane and its derived carbon membranes. J. Membr. Sci..

[23] Fu Y-J, Hu C-C, Lee K-R, Lai J-Y (2008). Effects of residual solvent on gas separation properties of polyimide membranes. Sep. Purif. Technol..

[24] Joly C, Le Cerf D, Chappey C, Muller G (1999). Residual solvent effect on the permeation properties of fluorinated polyimide films. Sep. Purif. Technol..

[25] Penkova A, Polotskaya G, Toikka A, Kocherbitov V (2011). Effect of Residual Solvent on Physicochemical Properties of Poly(Phenylene Isophtalamide) Membrane. Drying Technol..

[26] Pulyalina AY, Toikka AM, Polotskaya GA (2014). Investigation of Pervaporation Membranes Based on Polycarbamide: Effect of Residual Solvent. Petroleum Chemistry.

[27] Kim J-H, Lee K-H, Kim SY (2000). Pervaporation separation of water from ethanol through polyimide composite membranes. J. Membr. Sci..

[28] Yanagisita H (1997). Preparation and pervaporation performance of polyimide composite membrane by vapor deposition and polymerization (VDP). J. Membr. Sci..

[29] Liu Y-L, Yu C-H, Lai J-Y (2008). Poly(tetrafluoroethylene)/polyamide thin-film composite membranes via interfacial polymerization for pervaporation dehydration on an isopropanol aqueous solution. J. Membr. Sci..

[30] Li C-L, Huang S-H, Liaw D-J, Lee K-R, Lai J-Y (2008). Interfacial polymerized thin-film composite membranes for pervaporation separation of aqueous isopropanol solution. Sep. Purif. Technol..

[31] Kocherbitov V, Ulvenlund S, Kober M, Jarring K, Arnebrant T (2008). Hydration of Microcrystalline Cellulose and Milled Cellulose Studied by Sorption Calorimetry. J. Phys. Chem. B.

[32] Kocherbitov V, Arnebrant T, Soderman O (2004). Lysozyme−Water Interactions Studied by Sorption Calorimetry. J. Phys. Chem. B.

[33] Kocherbitov V, Söderman O (2004). Glassy Crystalline State and Water Sorption of Alkyl Maltosides. Langmuir.

[34] Polotskaya GA, Kostereva TA, Elyashevich GK (1998). Gas transport properties and structural order of poly(4,4′-oxydiphenylene piromelliteimide) in composite membranes. Sep. Purif. Technol..

[35] Polotskaya GA, Sklizkova VP, Kozhurnikova ND, Elyashevich GK, Kudryavtsev VV (2000). Formation and analysis of a polyimide layer in composite membranes. J. Appl. Polym. Sci..

[36] Polotskaya GA, Arganova SA, Antonova TA, Elyashevich GK (1997). Polyphenylene oxide sulfonate-based composite membrane for gas-separation. Russ. J. Appl. Chem..

[37] Pulyalina AY (2010). Pervaporation membranes based on composites of polyimide with polyaniline or its copolymer. Desalin. Water Treat..

[38] Babalou, A.A., Rafia, N. & Ghasemzadeh, K. Pervaporation, Vapour Permeation and Membrane Distillation in *Pervaporation, Vapour Permeation and Membrane Distillation*. 459 (Elsevier, 2015).

[39] Xu YM, Le NL, Zuo J, Chung TS (2016). Aromatic polyimide and crosslinked thermally rearranged poly(benzoxazole-co-imide) membranes for isopropanol dehydration via pervaporation. J. Memb. Sci..

[40] Han YJ, Wang KH, Lai JY, Liu YL (2014). Hydrophilic chitosan-modified polybenzoimidazole membranes for pervaporation dehydration of isopropanol aqueous solutions. J. Memb. Sci..

[41] Xiao S, Feng X, Huang RYM (2008). 2,2-Bis[4-(3,4-Dicarboxyphenoxy) Phenyl]Propane Dianhydride (BPADA)-Based Polyimide Membranes for Pervaporation Dehydration of Isopropanol: Characterization and Comparison with 4,40-(Hexafluoroisopropylidene) Diphthalic Anhydride (6FDA) -Based Polyimide Membranes. J. Appl. Polym. Sci..

[42] Li Y, Li W, Zhang Y, Liu D (2005). Studies on the synthesis of 4,4-diaminodiphenylurea and direct dyes derived therefrom. Dyes and Pigments.

[43] Goikhman MY (1997). Synthesis and properties of polybenzoxazinonimedes. Polymer Science A.

[44] Polotskaya GA (1992). Composite gas separation membrane based on polyamidoimide-poly-2,6 – dimethyl-1,4-polyphenylenoxide. Polymer Science A.

[45] Polotsky AE, Polotskaya GA (1998). Study on top layer structure of composite membranes. J. Membr. Sci..

[46] Andreev GA, Hartmanoá M (1989). Flotation method of precise density measurements. Phys. Stat. Sol. A..

[47] Chalykh AE (2001). Diffusion – method of polymer system investigation. Polymer Science A.

[48] Askadskii, А.А., Matveev, Y.B The chemical structure and physical properties of polymers (Chemistry, Moscow, USSR, 1983).

[49] Tager, A.A. Physical chemistry of polymers (Nauchnyi mir 2007).

[50] Wadso I, Wadso L (1996). A new method for determination of vapour sorption isotherms using a twin double microcalorimeter. Thermochim. Acta..

[51] Kocherbitov V (2004). A new formula for accurate calculation of water activity in sorption calorimetric experiments. Thermochim. Acta..

[52] Pulyalina A (2013). Study on polybenzoxazinone membrane in pervaporation processes. J. Appl. Polym. Sci..

[53] Kittur AA, Kariduraganavar MY, Toti US, Ramesh K, Aminabhavi TM (2003). Pervaporation separation of water–isopropanol mixtures using ZSM-5 zeolite incorporated poly(vinyl alcohol) membranes. J. Appl. Polym. Sci..

[54] Lee YM, Bourgeois D, Belfort G (1989). Sorption, diffusion, and pervaporation of organics in polymer membranes. J. Membr. Sci..

[55] Fagerstrom A (2013). Effects of surfactants and thermodynamic activity of model active ingredient on transport over plant leaf cuticle. Colloids Surf., B.

[56] Baker RW, Wijmans JG, Huang Y (2010). Permeability, permeance and selectivity: A preferred way of reporting pervaporation performance data. J. Membr. Sci..

